# Supplementary figures and images for: GJA1 depletion causes ciliary defects by affecting Rab11 trafficking to the ciliary base
Source: eLife. 2022 Aug 25;11:e81016. doi: 10.7554/eLife.81016 (PMC9448326; doi:10.7554/eLife.81016)

## Slide 1
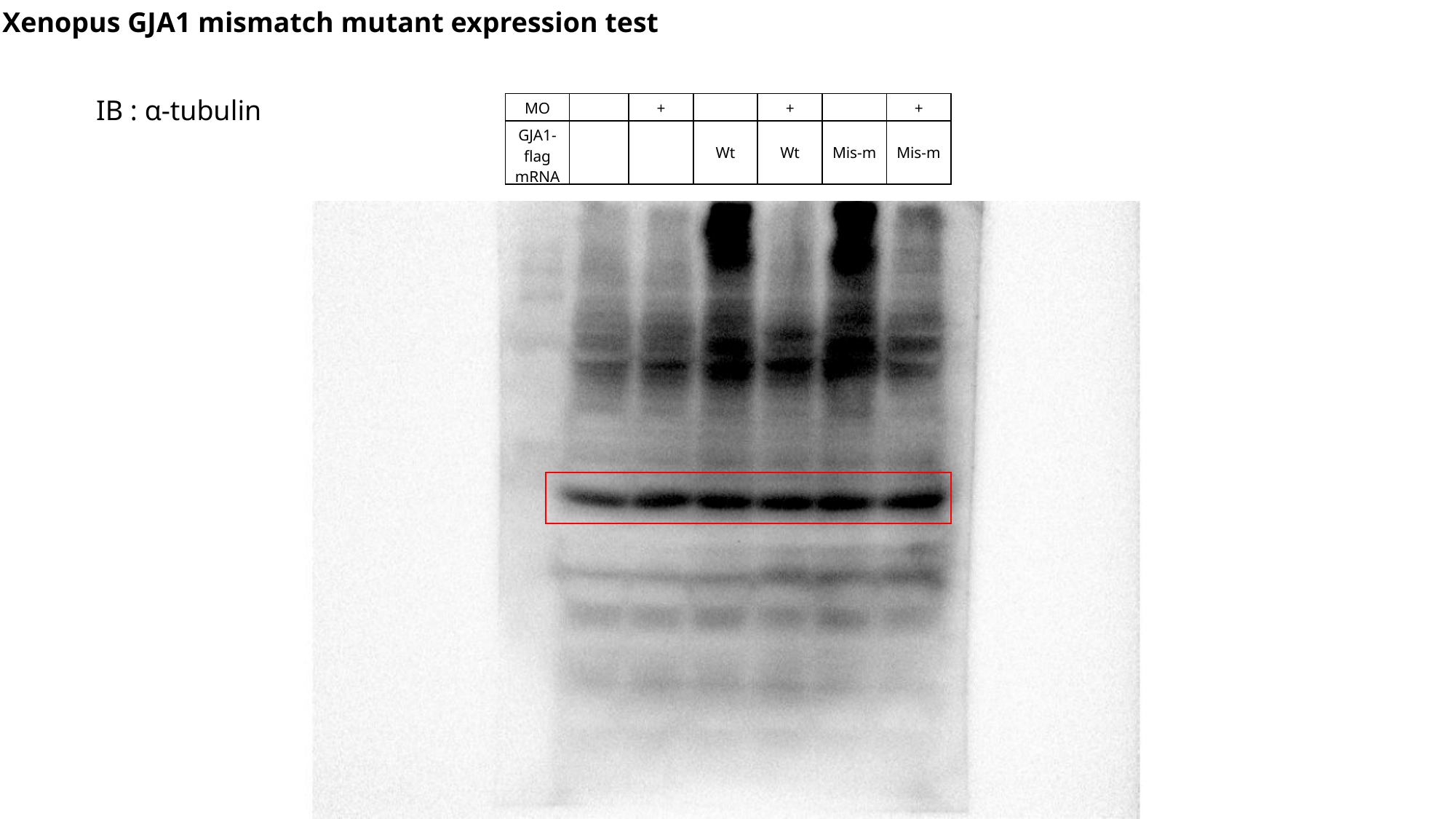

Xenopus GJA1 mismatch mutant expression test
IB : α-tubulin
| MO | | + | | + | | + |
| --- | --- | --- | --- | --- | --- | --- |
| GJA1-flag mRNA | | | Wt | Wt | Mis-m | Mis-m |

Supplement: Figure 3—source data 2. [file elife-81016-fig3-data2.zip › alpha-tubulin-Western blot/Figure.pptx]

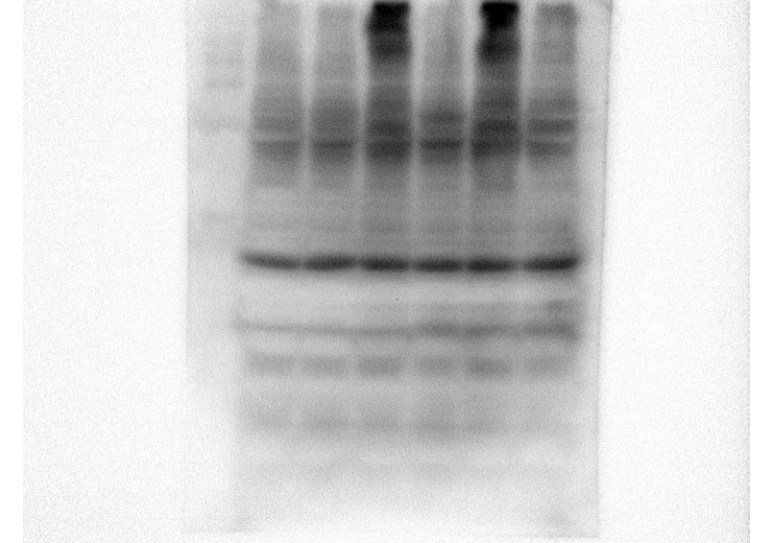

Supplement: Figure 3—source data 2. [file elife-81016-fig3-data2.zip › alpha-tubulin-Western blot/Raw file.tif]

## Slide 1
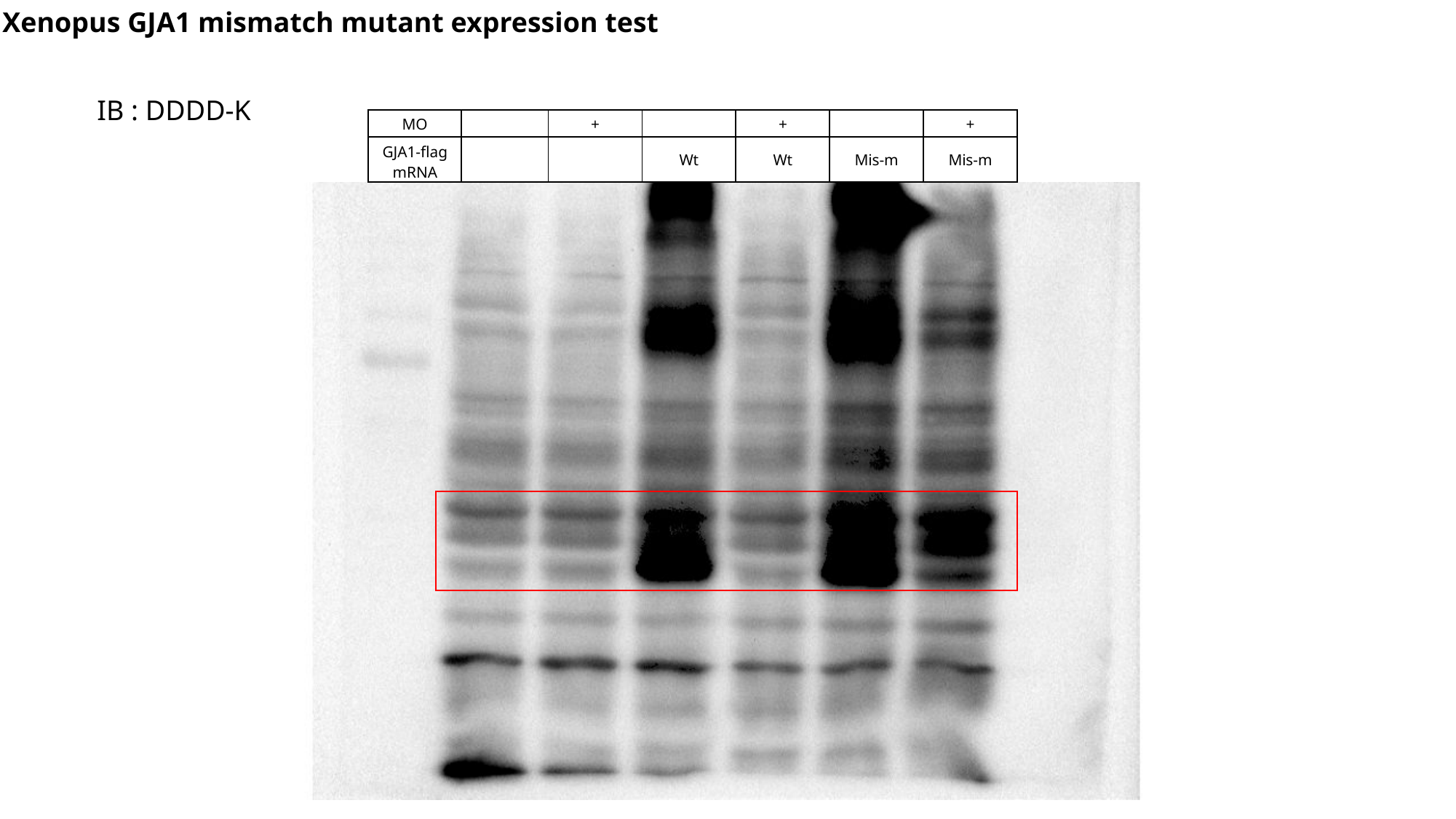

Xenopus GJA1 mismatch mutant expression test
IB : DDDD-K
| MO | | + | | + | | + |
| --- | --- | --- | --- | --- | --- | --- |
| GJA1-flag mRNA | | | Wt | Wt | Mis-m | Mis-m |

Supplement: Figure 3—source data 2. [file elife-81016-fig3-data2.zip › GJA1-Flag-Western blot/Figure.pptx]

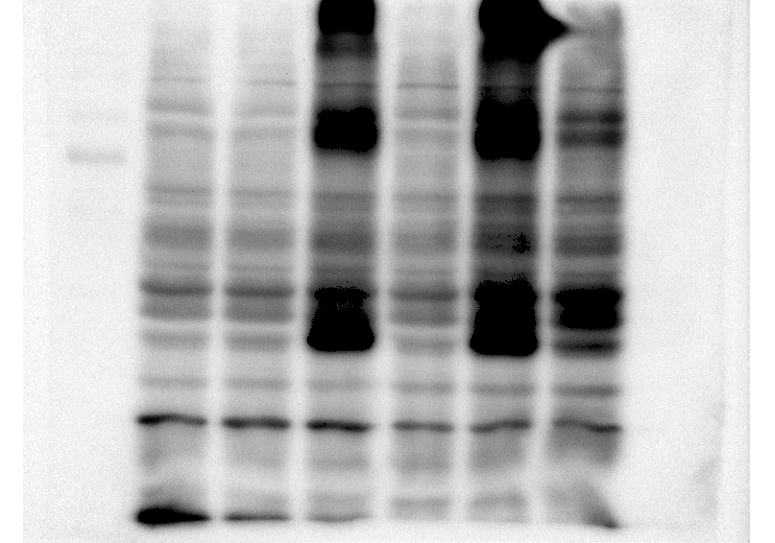

Supplement: Figure 3—source data 2. [file elife-81016-fig3-data2.zip › GJA1-Flag-Western blot/Raw file.tif]

## Slide 1
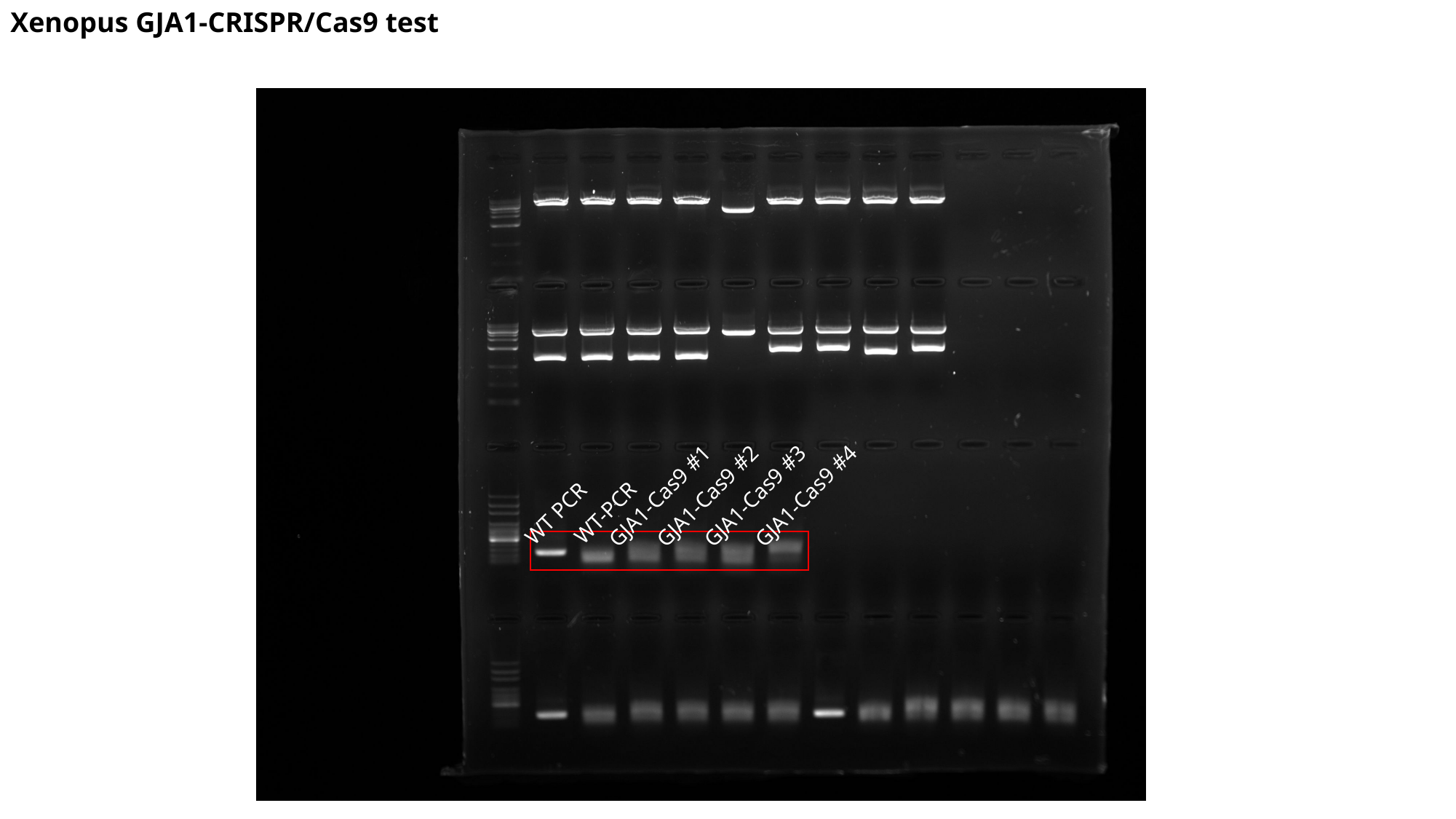

Xenopus GJA1-CRISPR/Cas9 test
GJA1-Cas9 #1
GJA1-Cas9 #2
GJA1-Cas9 #3
GJA1-Cas9 #4
WT-PCR
WT PCR

Supplement: Figure 3—figure supplement 2—source data 2. [file elife-81016-fig3-figsupp2-data2.zip › Figure.pptx]

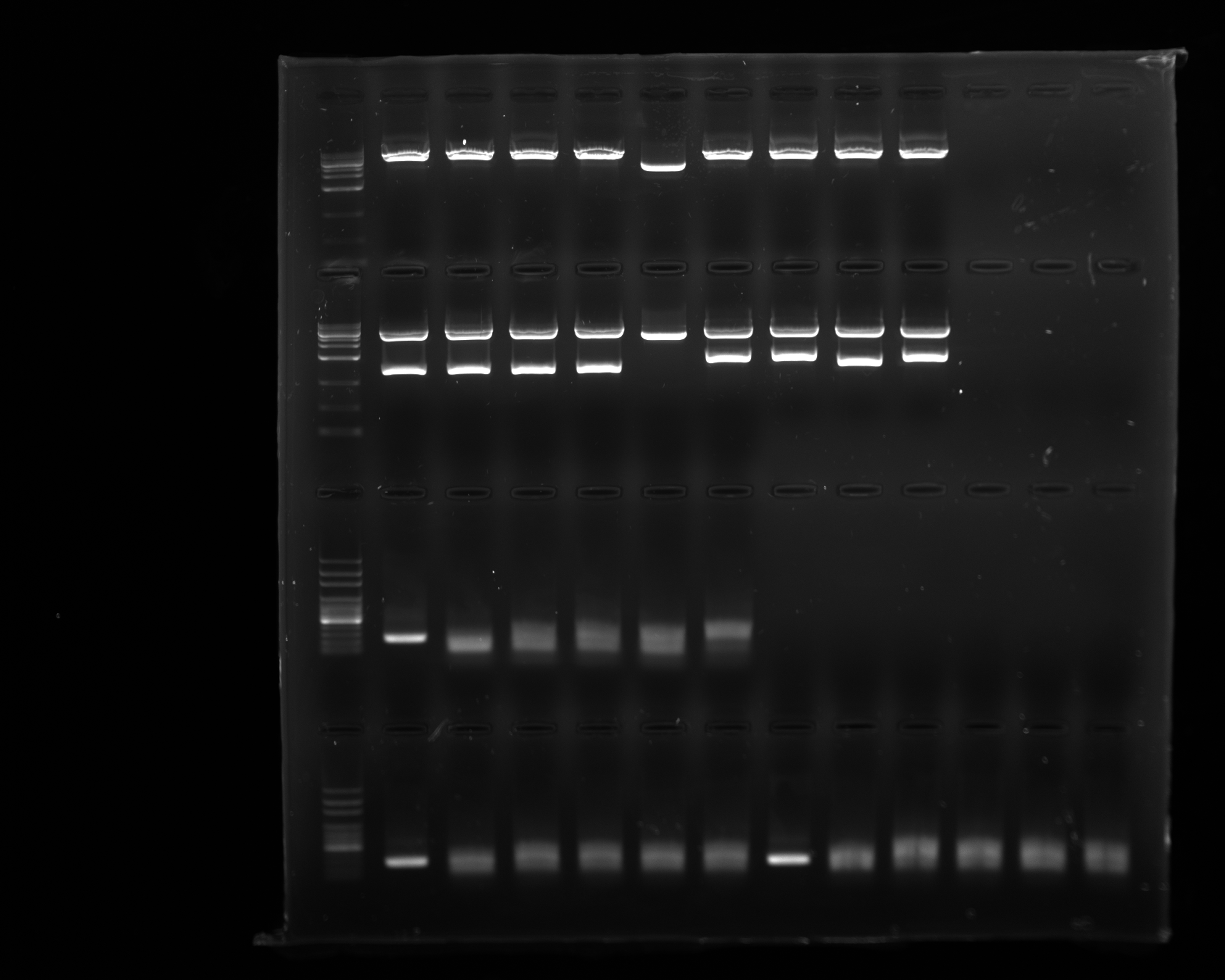

Supplement: Figure 3—figure supplement 2—source data 2. [file elife-81016-fig3-figsupp2-data2.zip › Raw file.tif]

## Slide 1
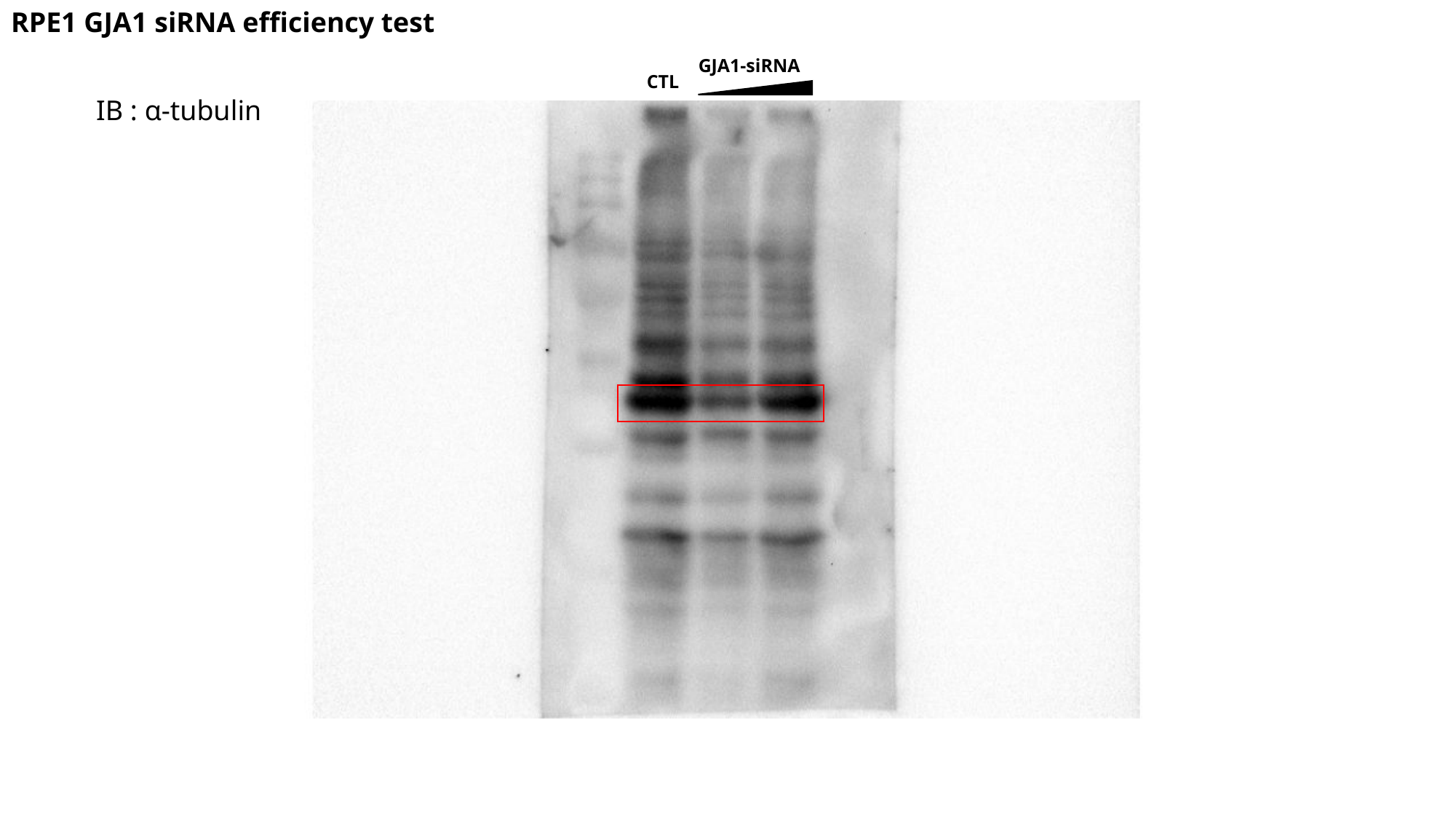

RPE1 GJA1 siRNA efficiency test
GJA1-siRNA
CTL
IB : α-tubulin

Supplement: Figure 5—figure supplement 1—source data 2. [file elife-81016-fig5-figsupp1-data2.zip › alpha-tubulin-Western blot/Figure.pptx]

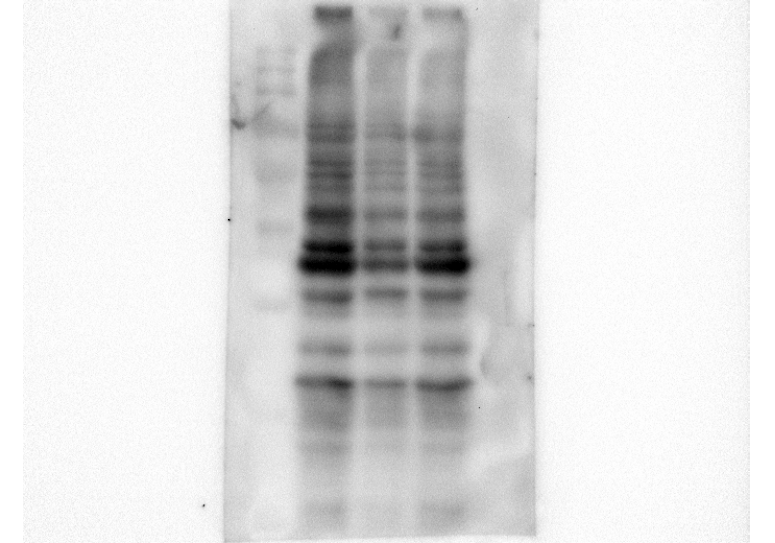

Supplement: Figure 5—figure supplement 1—source data 2. [file elife-81016-fig5-figsupp1-data2.zip › alpha-tubulin-Western blot/Raw file.tif]

## Slide 1
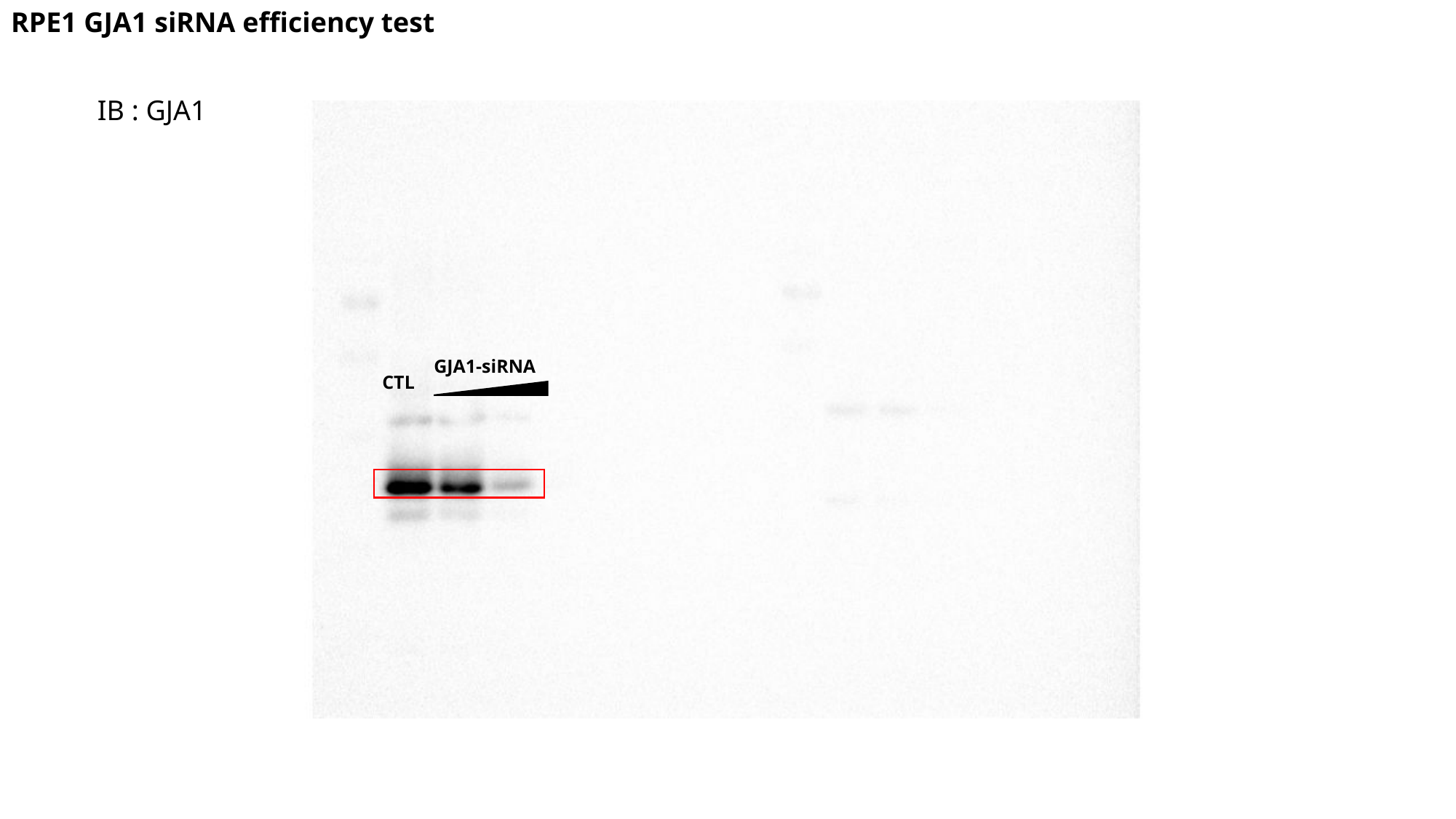

RPE1 GJA1 siRNA efficiency test
IB : GJA1
GJA1-siRNA
CTL

Supplement: Figure 5—figure supplement 1—source data 2. [file elife-81016-fig5-figsupp1-data2.zip › GJA1-Western blot/Figure.pptx]

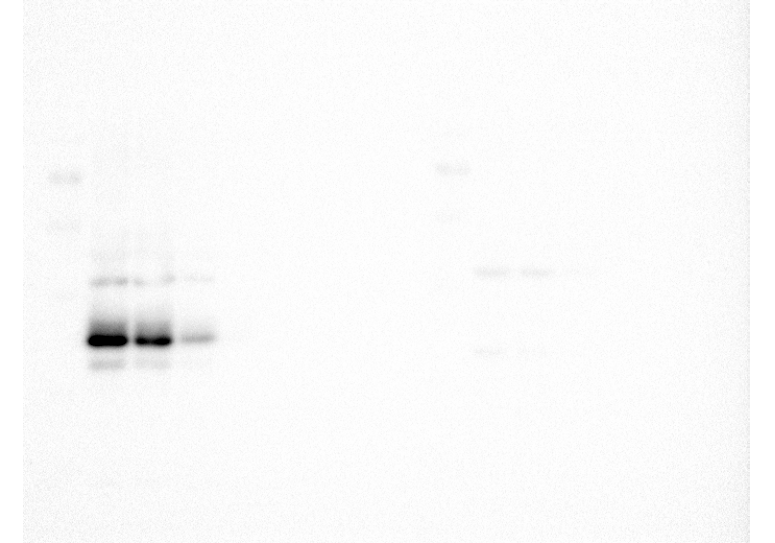

Supplement: Figure 5—figure supplement 1—source data 2. [file elife-81016-fig5-figsupp1-data2.zip › GJA1-Western blot/Raw file.tif]

## Slide 1
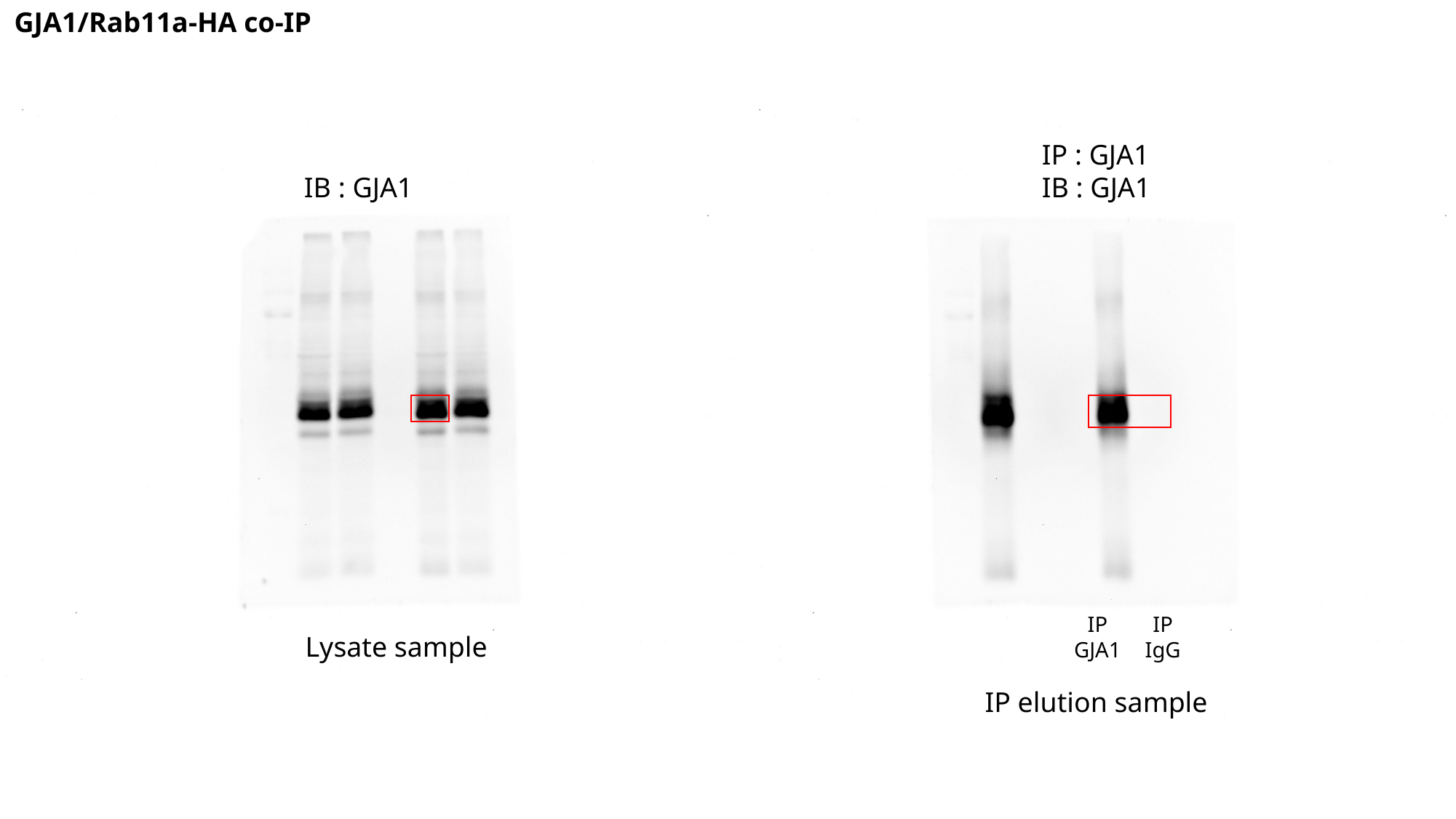

GJA1/Rab11a-HA co-IP
IP : GJA1
IB : GJA1
IB : GJA1
IP
GJA1
IP
IgG
Lysate sample
IP elution sample

Supplement: Figure 7—source data 2. [file elife-81016-fig7-data2.zip › GJA1-Western Blot/Figure.pptx]

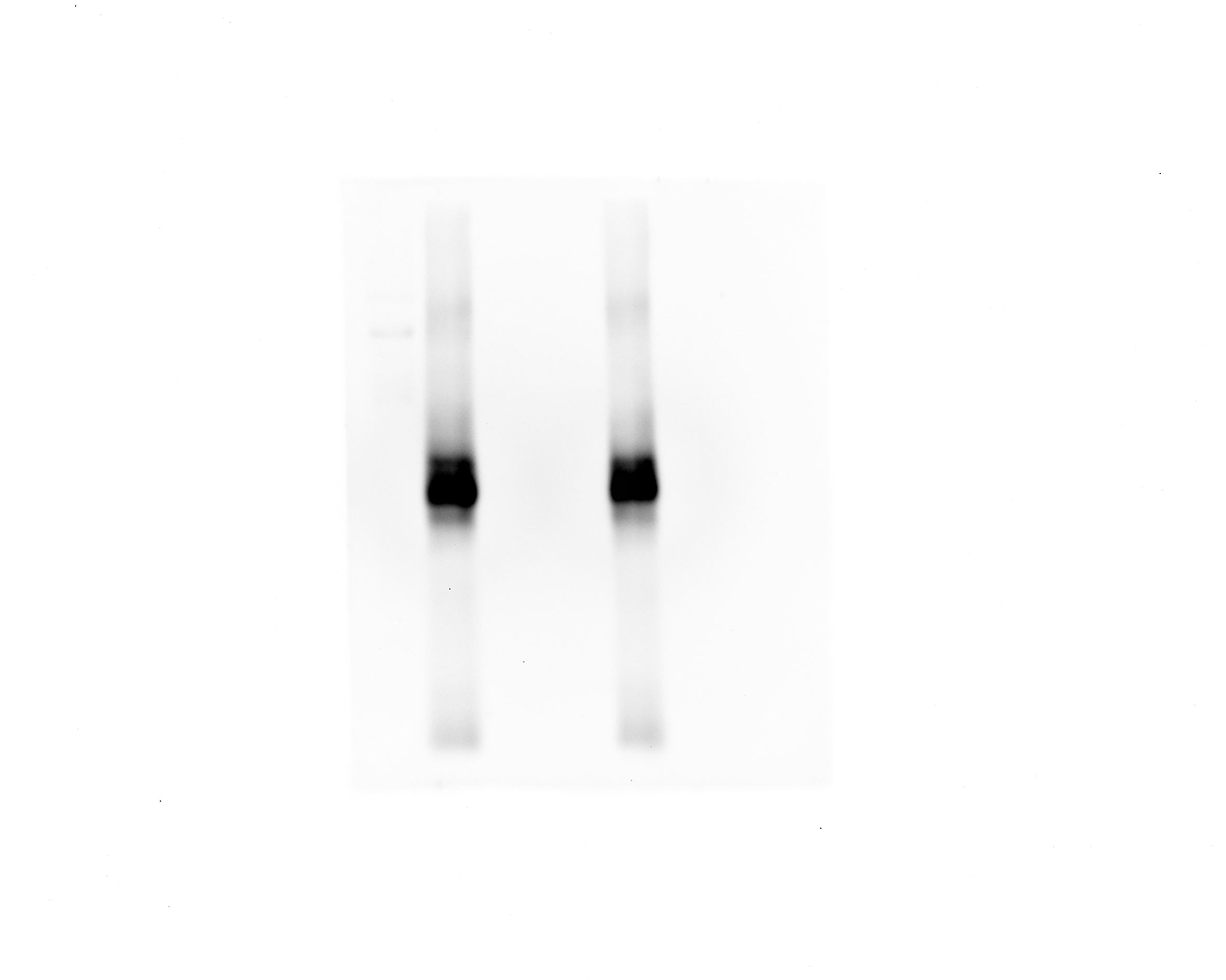

Supplement: Figure 7—source data 2. [file elife-81016-fig7-data2.zip › GJA1-Western Blot/IP Raw file.tif]

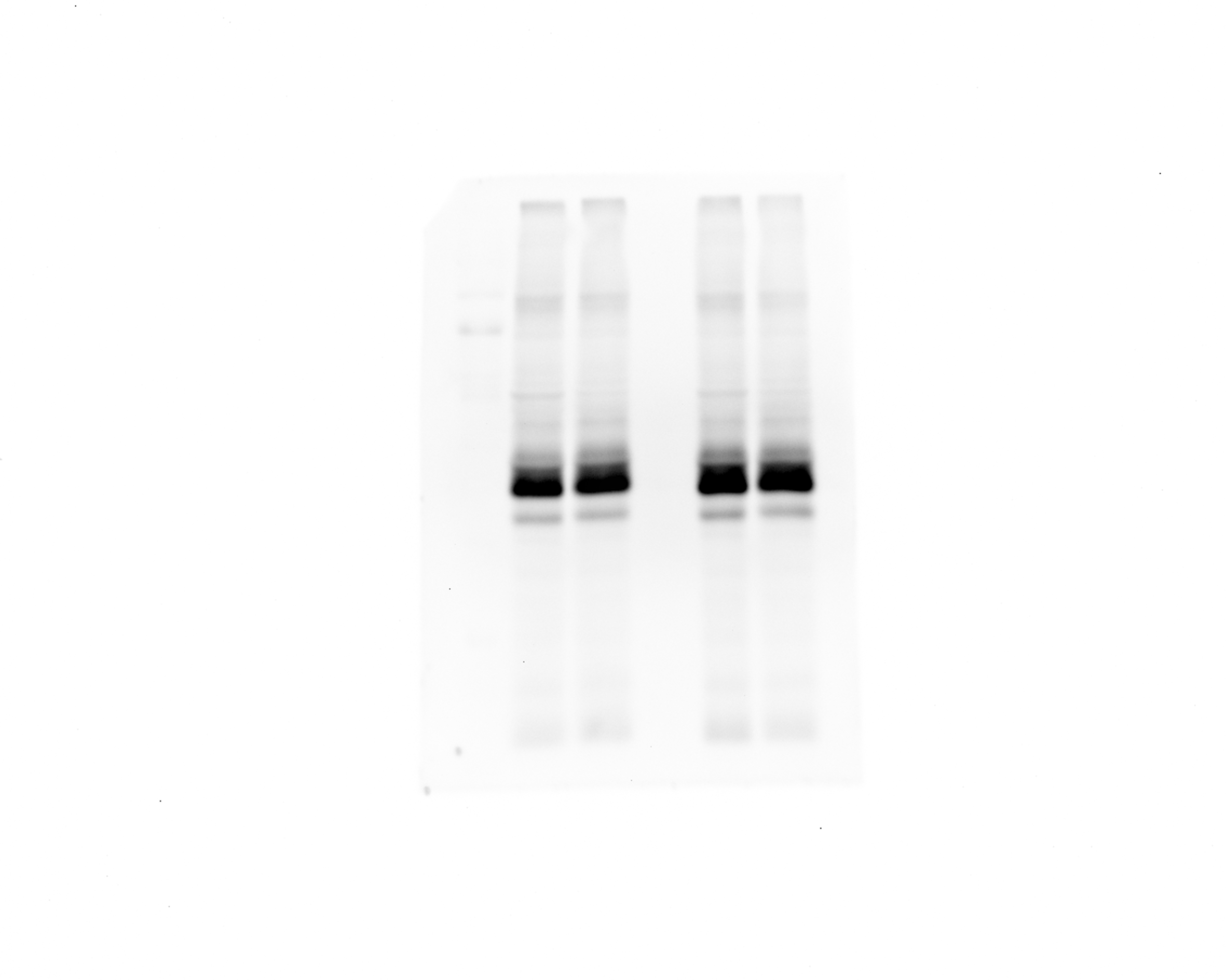

Supplement: Figure 7—source data 2. [file elife-81016-fig7-data2.zip › GJA1-Western Blot/Lysate Raw file.tif]

## Slide 1
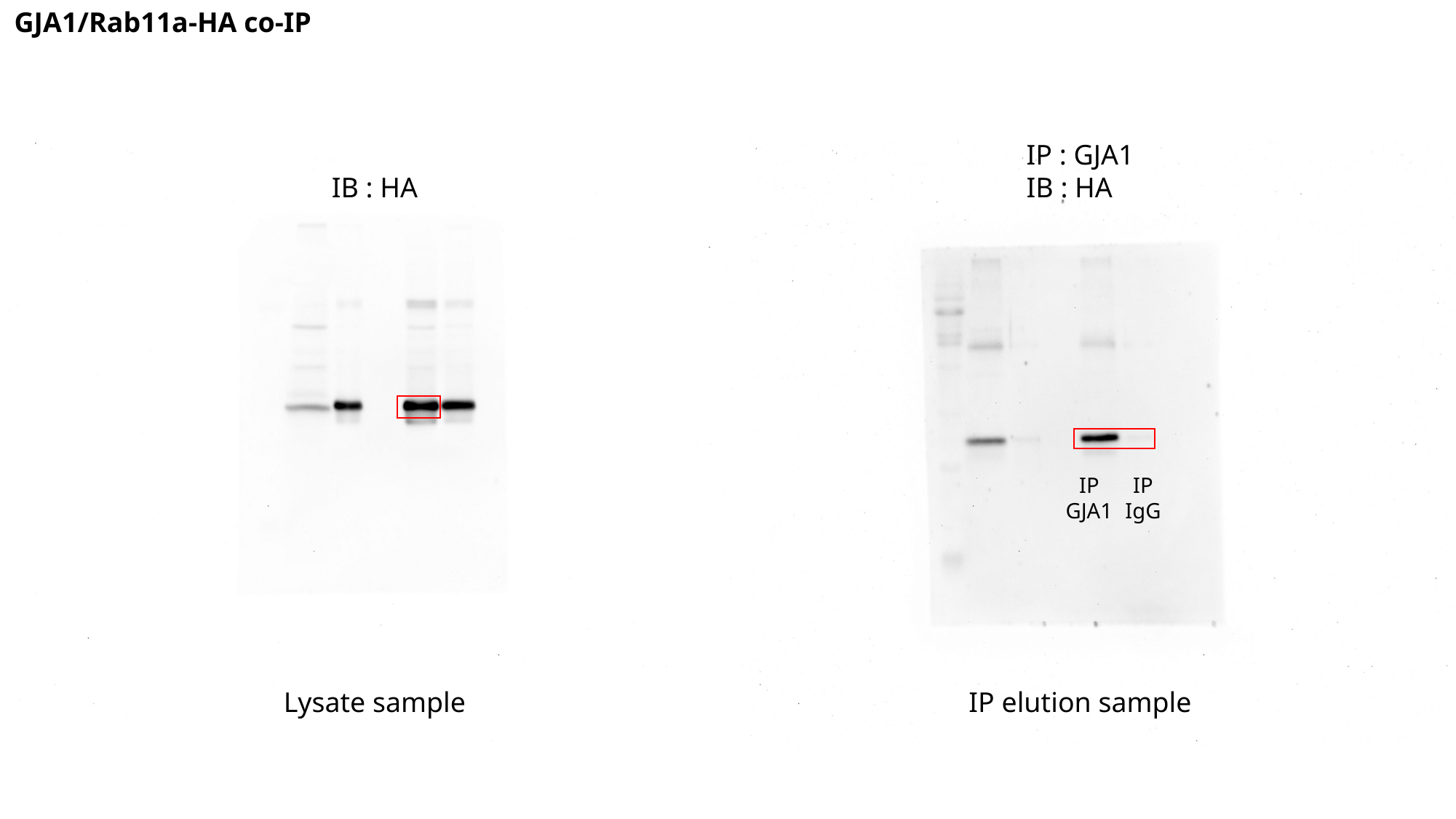

GJA1/Rab11a-HA co-IP
IP : GJA1
IB : HA
IB : HA
IP
GJA1
IP
IgG
Lysate sample
IP elution sample

Supplement: Figure 7—source data 2. [file elife-81016-fig7-data2.zip › Rab11a-HA-Western Blot/Figure.pptx]

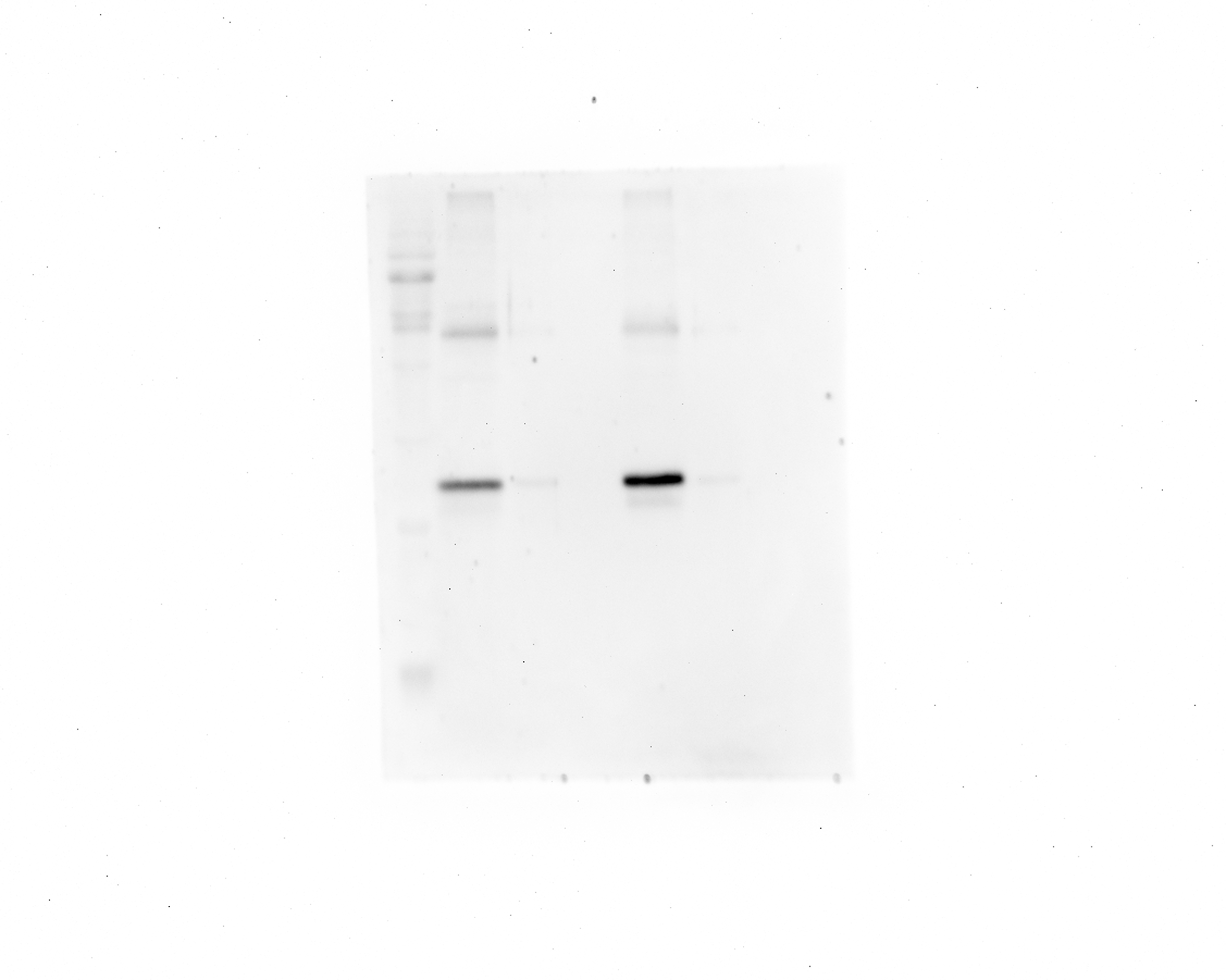

Supplement: Figure 7—source data 2. [file elife-81016-fig7-data2.zip › Rab11a-HA-Western Blot/IP Raw file.tif]

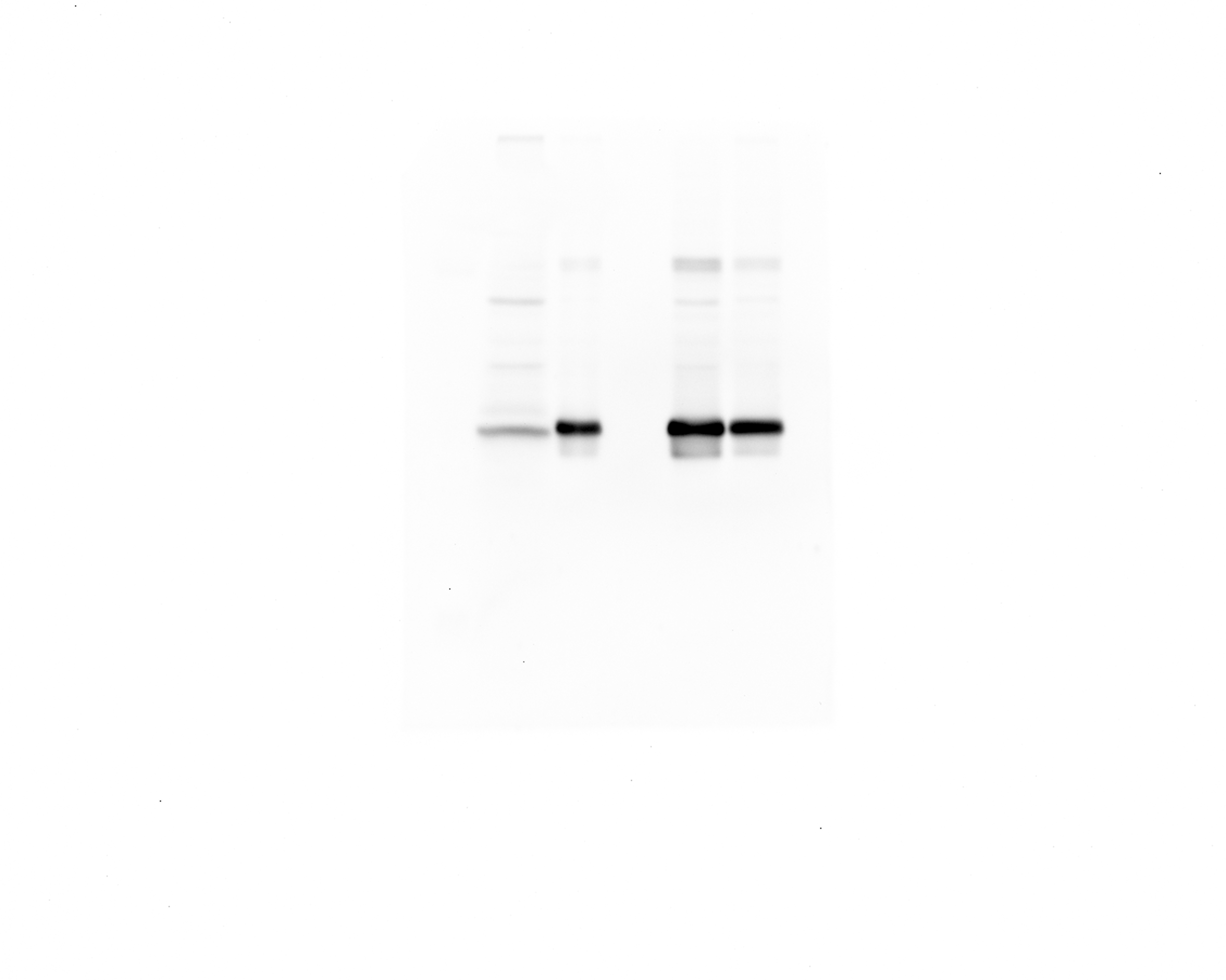

Supplement: Figure 7—source data 2. [file elife-81016-fig7-data2.zip › Rab11a-HA-Western Blot/Lysate Raw file.tif]

## Slide 1
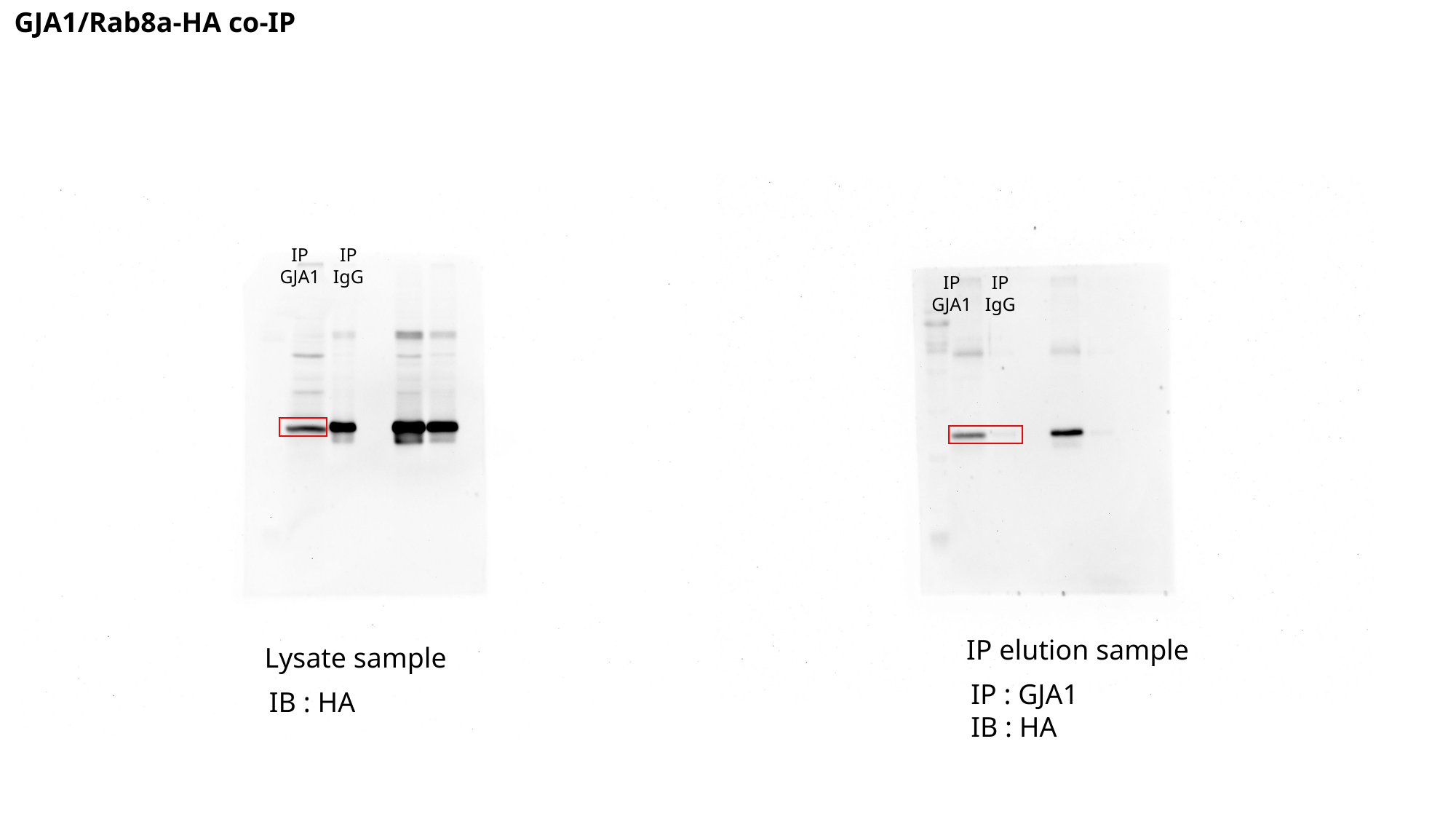

GJA1/Rab8a-HA co-IP
IP
GJA1
IP
IgG
IP
GJA1
IP
IgG
IP elution sample
Lysate sample
IP : GJA1
IB : HA
IB : HA

Supplement: Figure 7—figure supplement 3—source data 1. [file elife-81016-fig7-figsupp3-data1.zip › Rab8a-HA-Western blot/Figure.pptx]

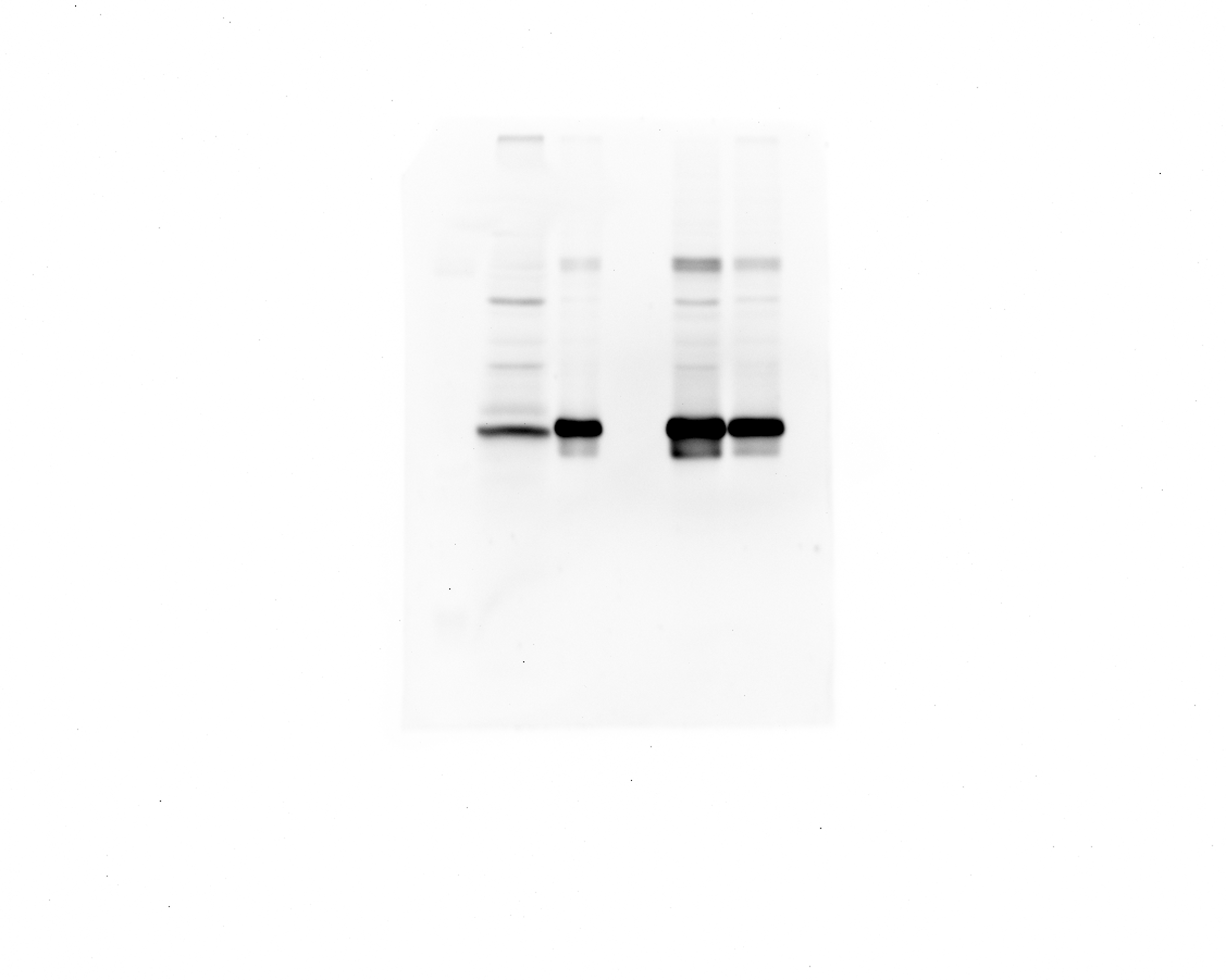

Supplement: Figure 7—figure supplement 3—source data 1. [file elife-81016-fig7-figsupp3-data1.zip › Rab8a-HA-Western blot/Lysate Raw file.tif]

## Slide 1
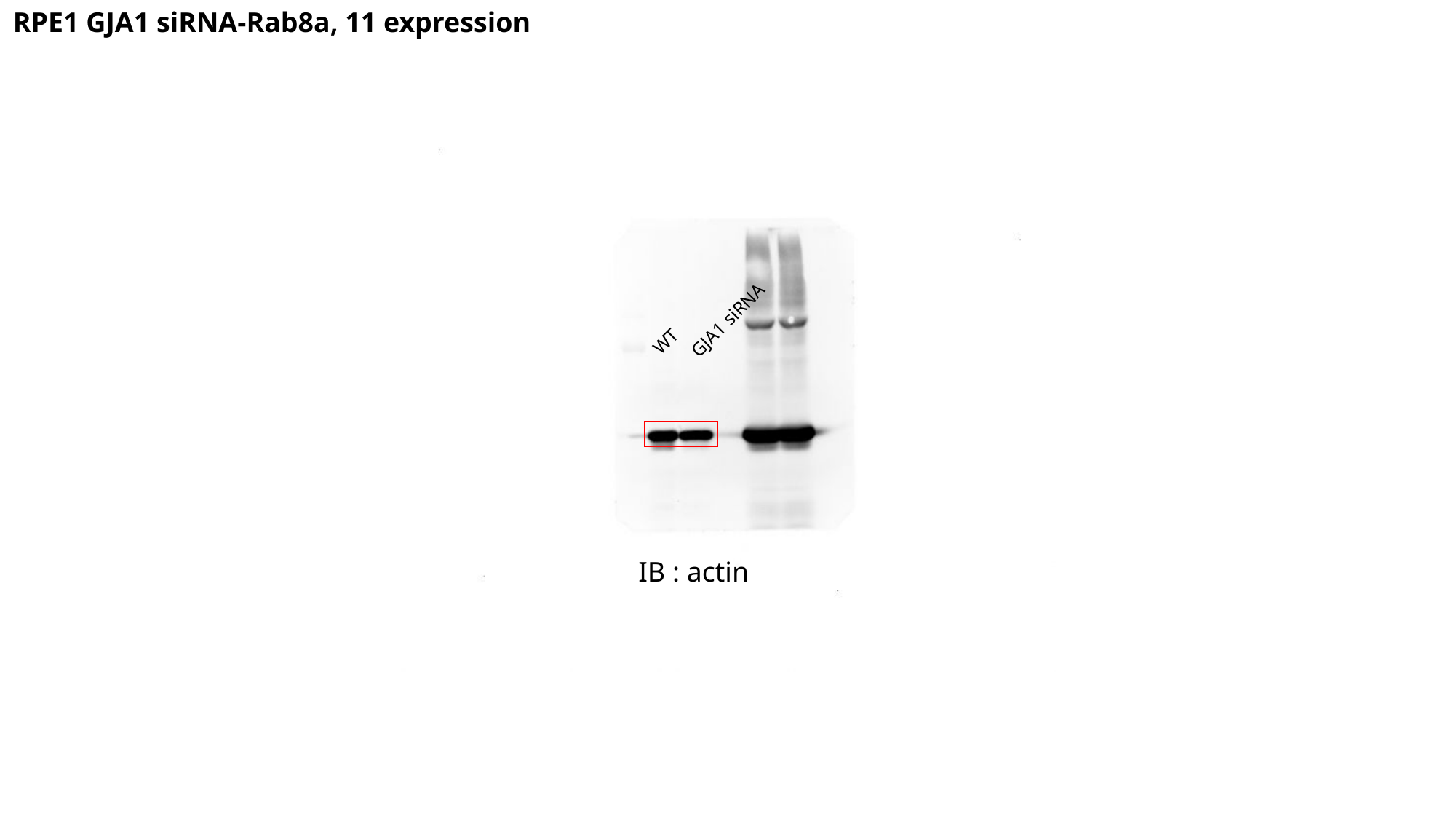

RPE1 GJA1 siRNA-Rab8a, 11 expression
GJA1 siRNA
WT
IB : actin

Supplement: Figure 7—figure supplement 3—source data 2. [file elife-81016-fig7-figsupp3-data2.zip › actin-Wesern blot/Figure.pptx]

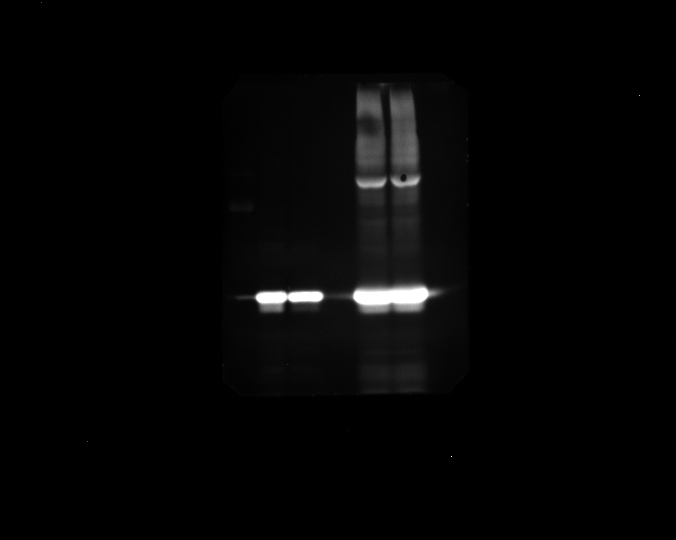

Supplement: Figure 7—figure supplement 3—source data 2. [file elife-81016-fig7-figsupp3-data2.zip › actin-Wesern blot/Raw file.tif]

## Slide 1
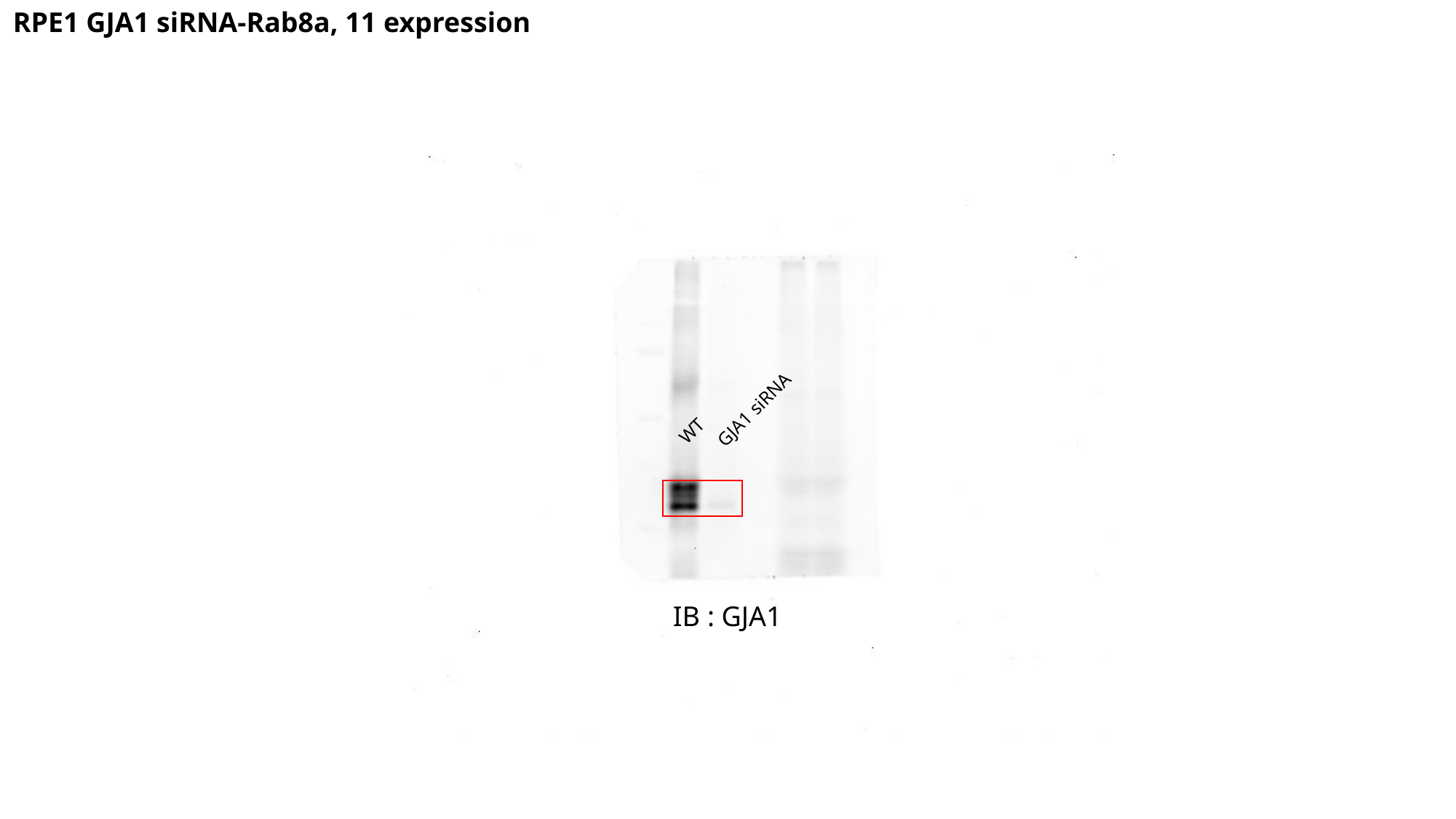

RPE1 GJA1 siRNA-Rab8a, 11 expression
GJA1 siRNA
WT
IB : GJA1

Supplement: Figure 7—figure supplement 3—source data 2. [file elife-81016-fig7-figsupp3-data2.zip › GJA1-Wesern blot/Figure.pptx]

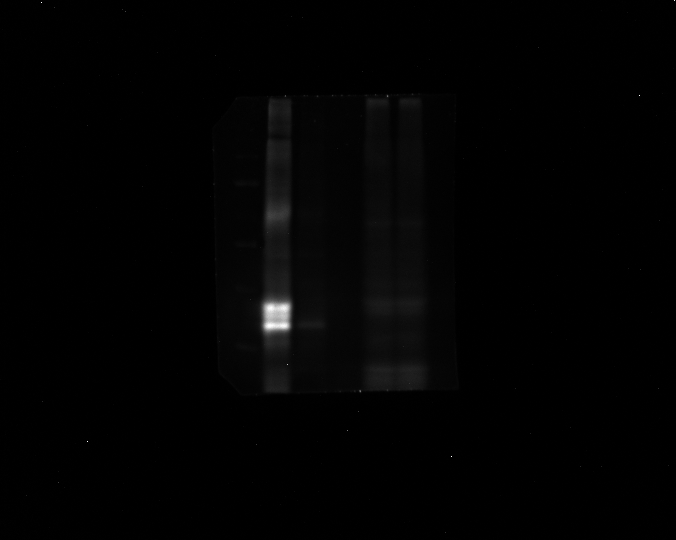

Supplement: Figure 7—figure supplement 3—source data 2. [file elife-81016-fig7-figsupp3-data2.zip › GJA1-Wesern blot/Raw file.tif]

## Slide 1
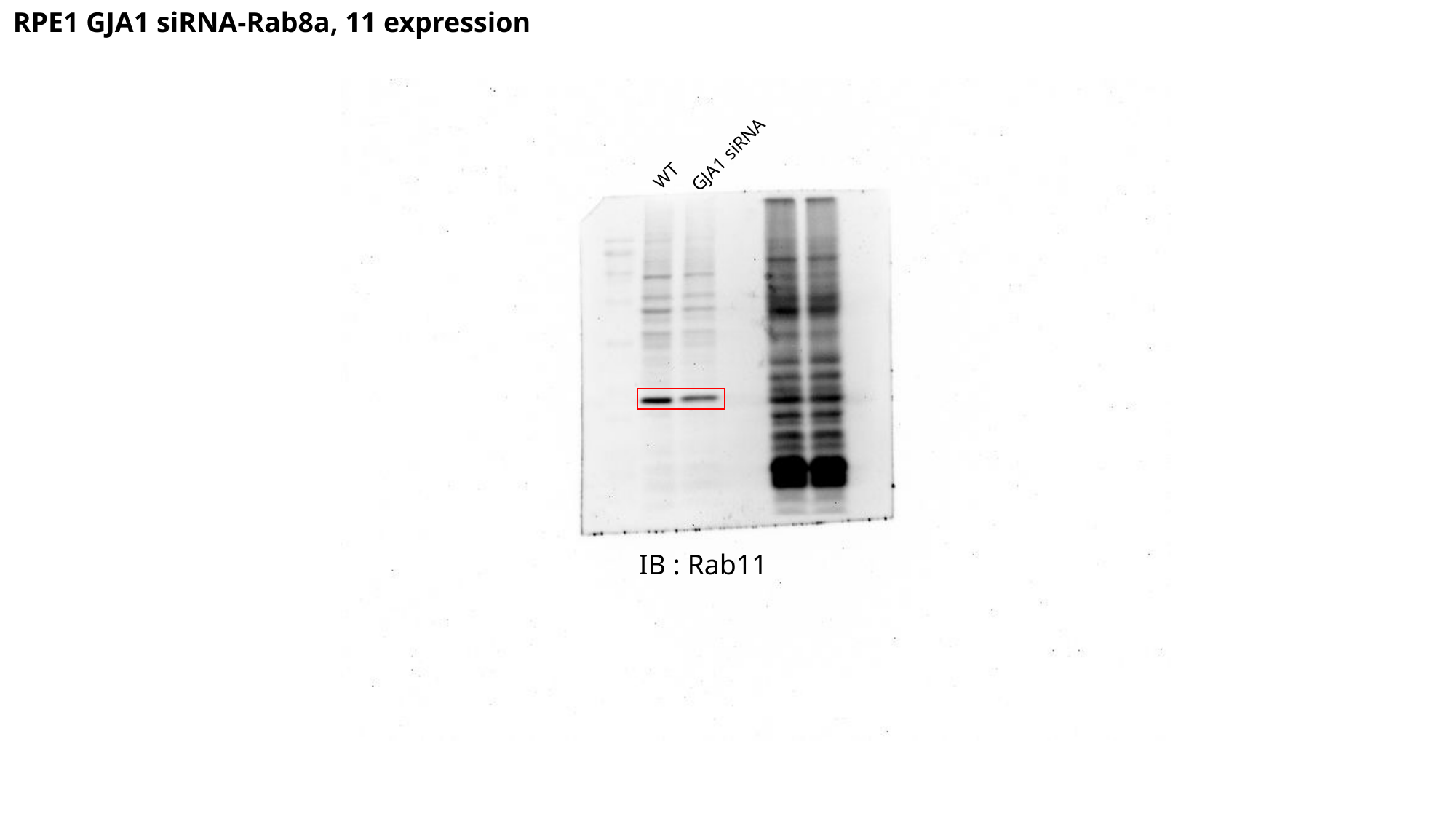

RPE1 GJA1 siRNA-Rab8a, 11 expression
GJA1 siRNA
WT
IB : Rab11

Supplement: Figure 7—figure supplement 3—source data 2. [file elife-81016-fig7-figsupp3-data2.zip › Rab11-Wesern blot/Figure.pptx]

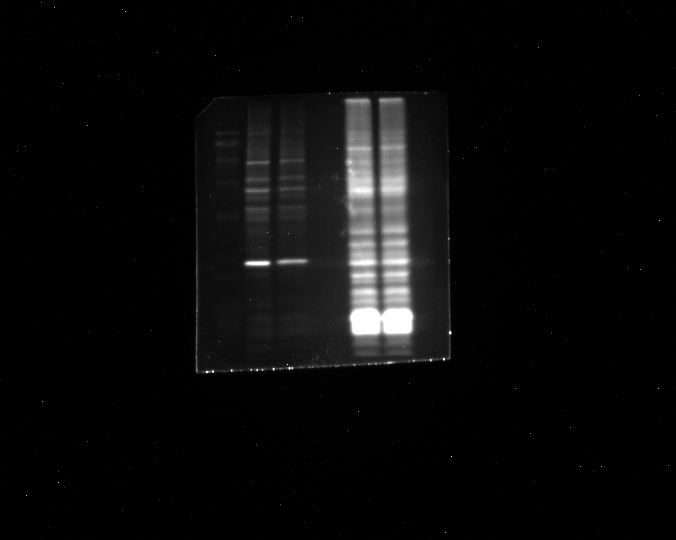

Supplement: Figure 7—figure supplement 3—source data 2. [file elife-81016-fig7-figsupp3-data2.zip › Rab11-Wesern blot/Raw file.tif]

## Slide 1
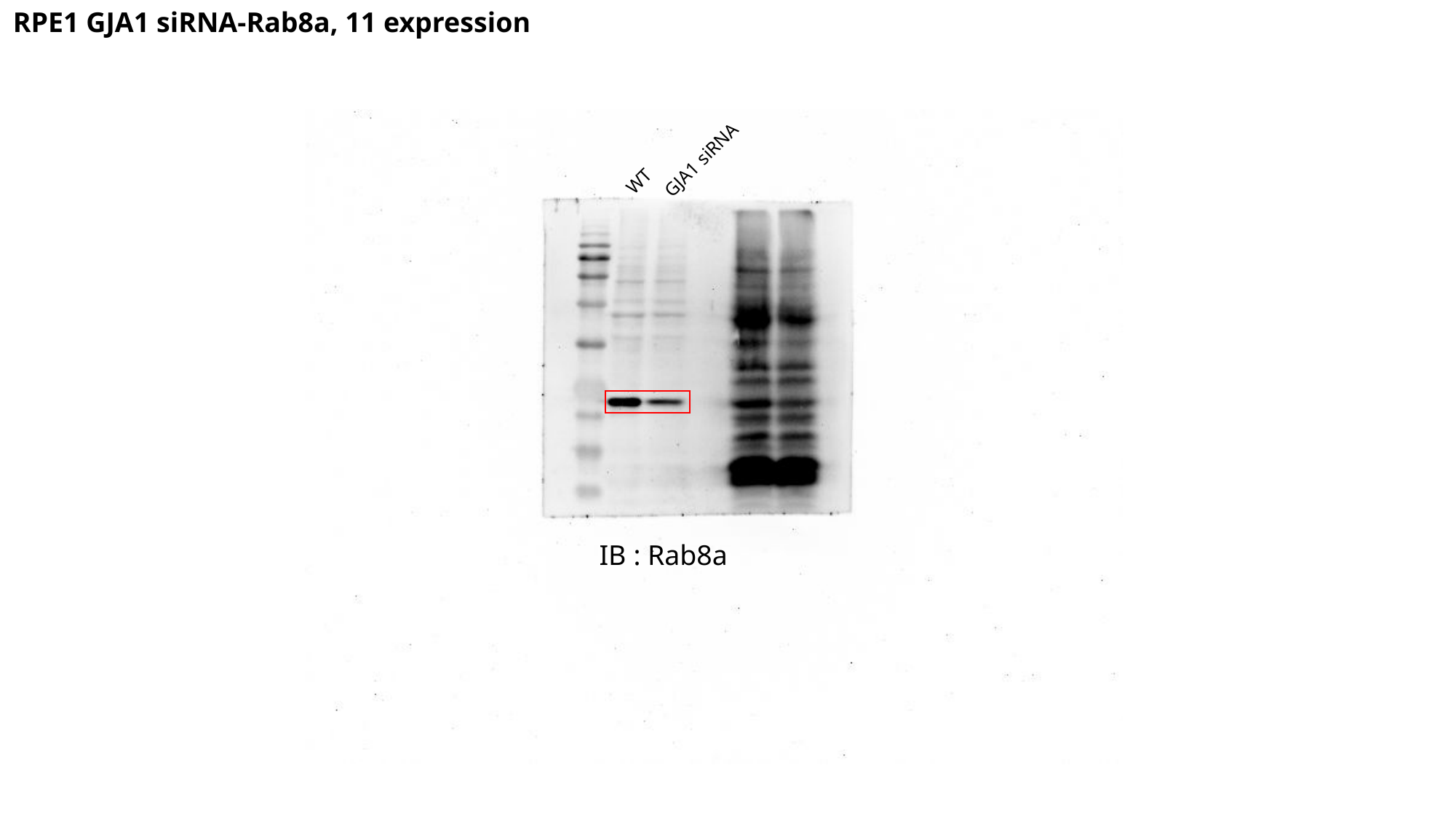

RPE1 GJA1 siRNA-Rab8a, 11 expression
GJA1 siRNA
WT
IB : Rab8a

Supplement: Figure 7—figure supplement 3—source data 2. [file elife-81016-fig7-figsupp3-data2.zip › Rab8a-Wesern blot/Figure.pptx]

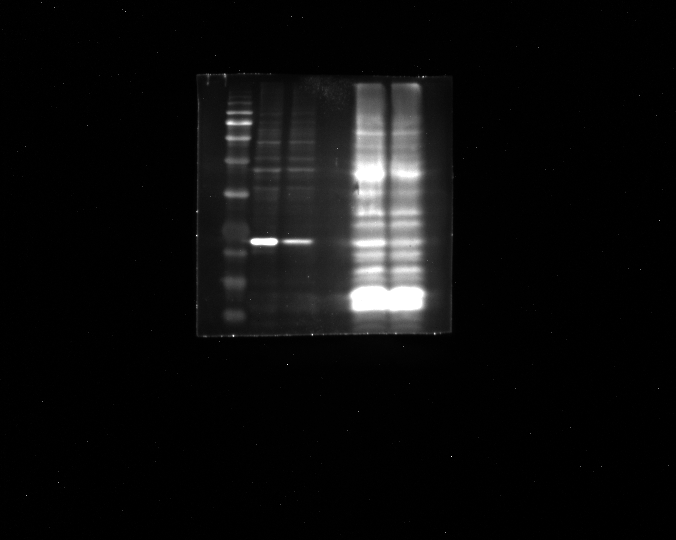

Supplement: Figure 7—figure supplement 3—source data 2. [file elife-81016-fig7-figsupp3-data2.zip › Rab8a-Wesern blot/Raw file.tif]

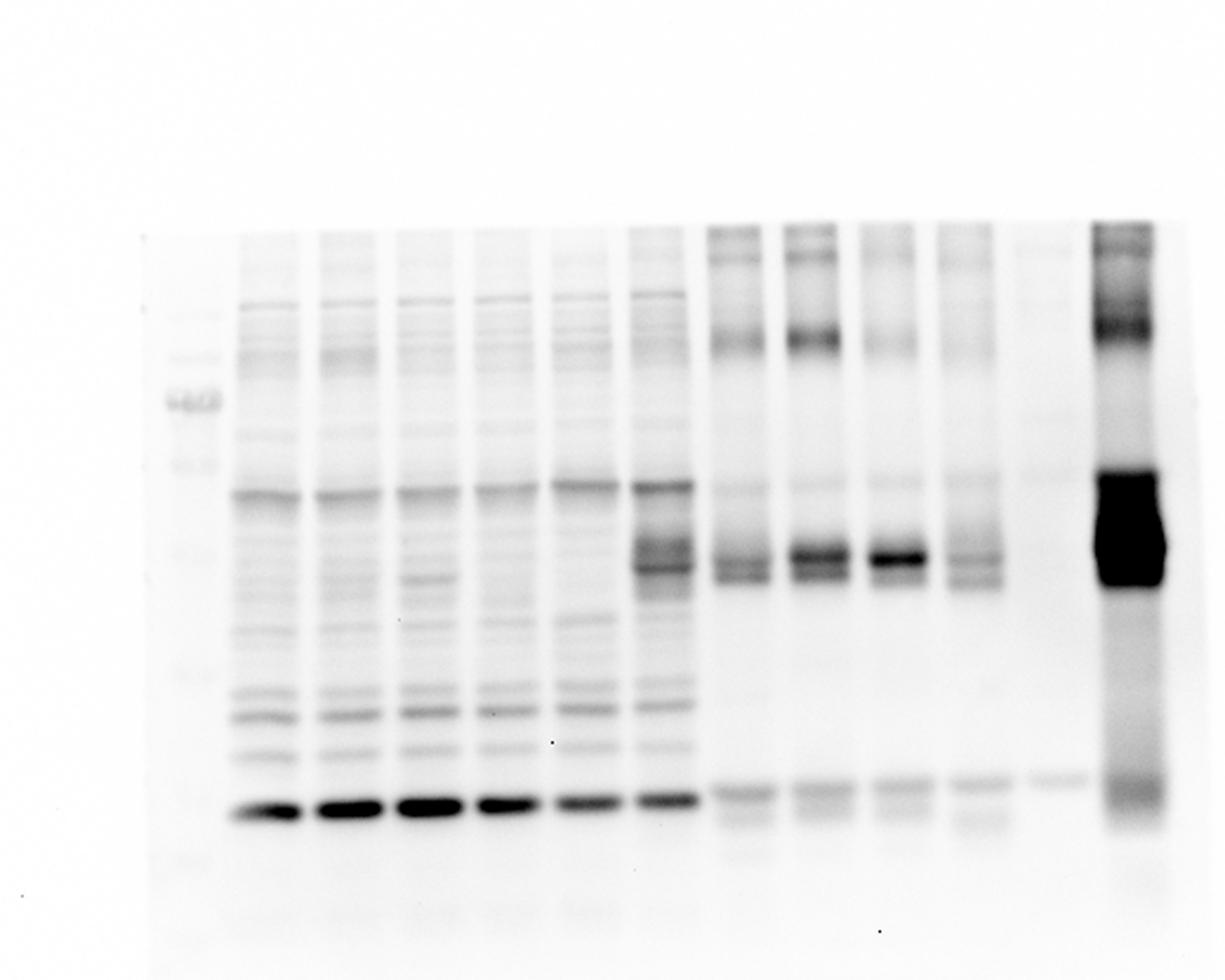

Supplement: Figure 8—source data 2. [file elife-81016-fig8-data2.zip › GJA1-Flag-Western blot/IP Raw file.png]

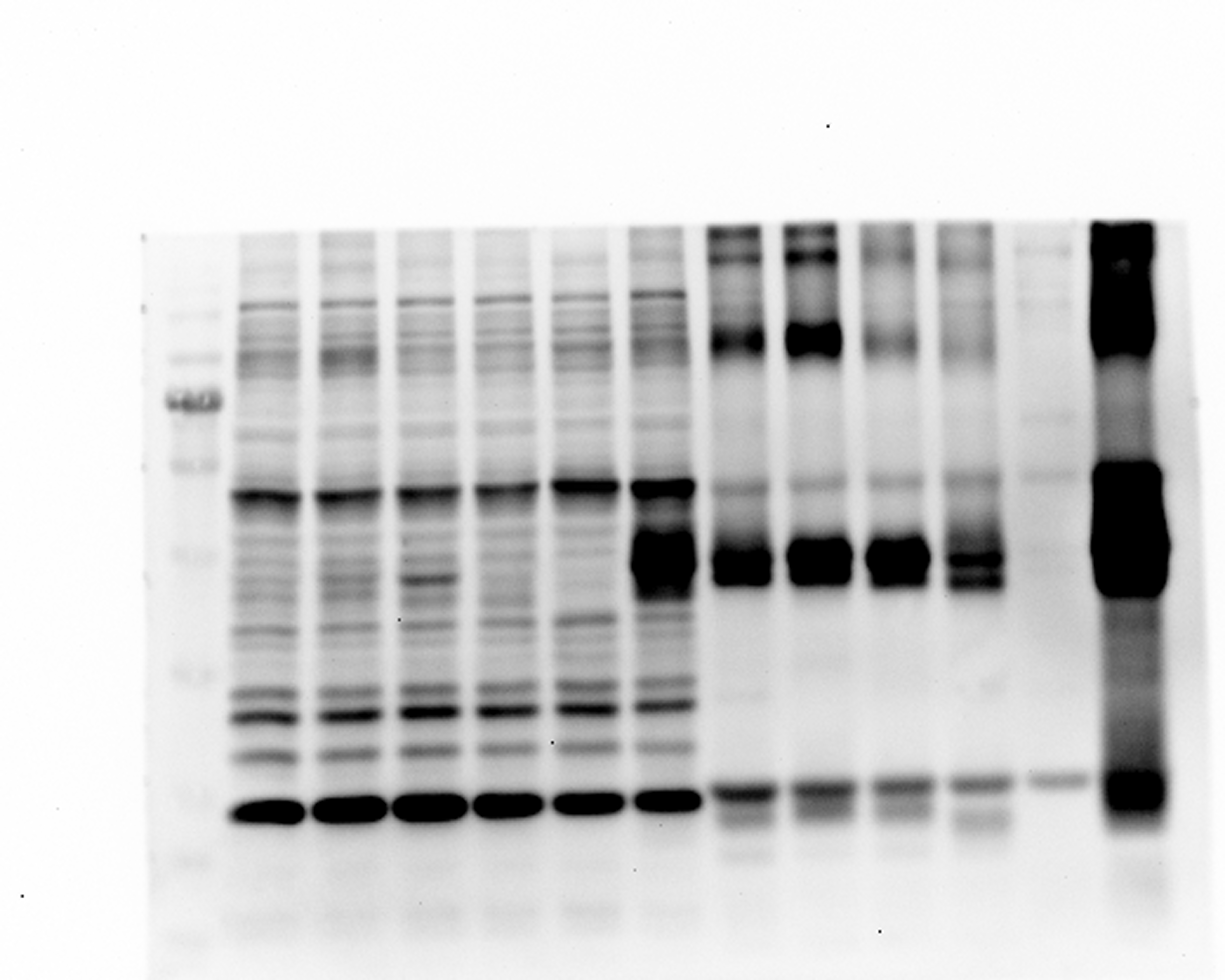

Supplement: Figure 8—source data 2. [file elife-81016-fig8-data2.zip › GJA1-Flag-Western blot/Lysate Raw file.png]

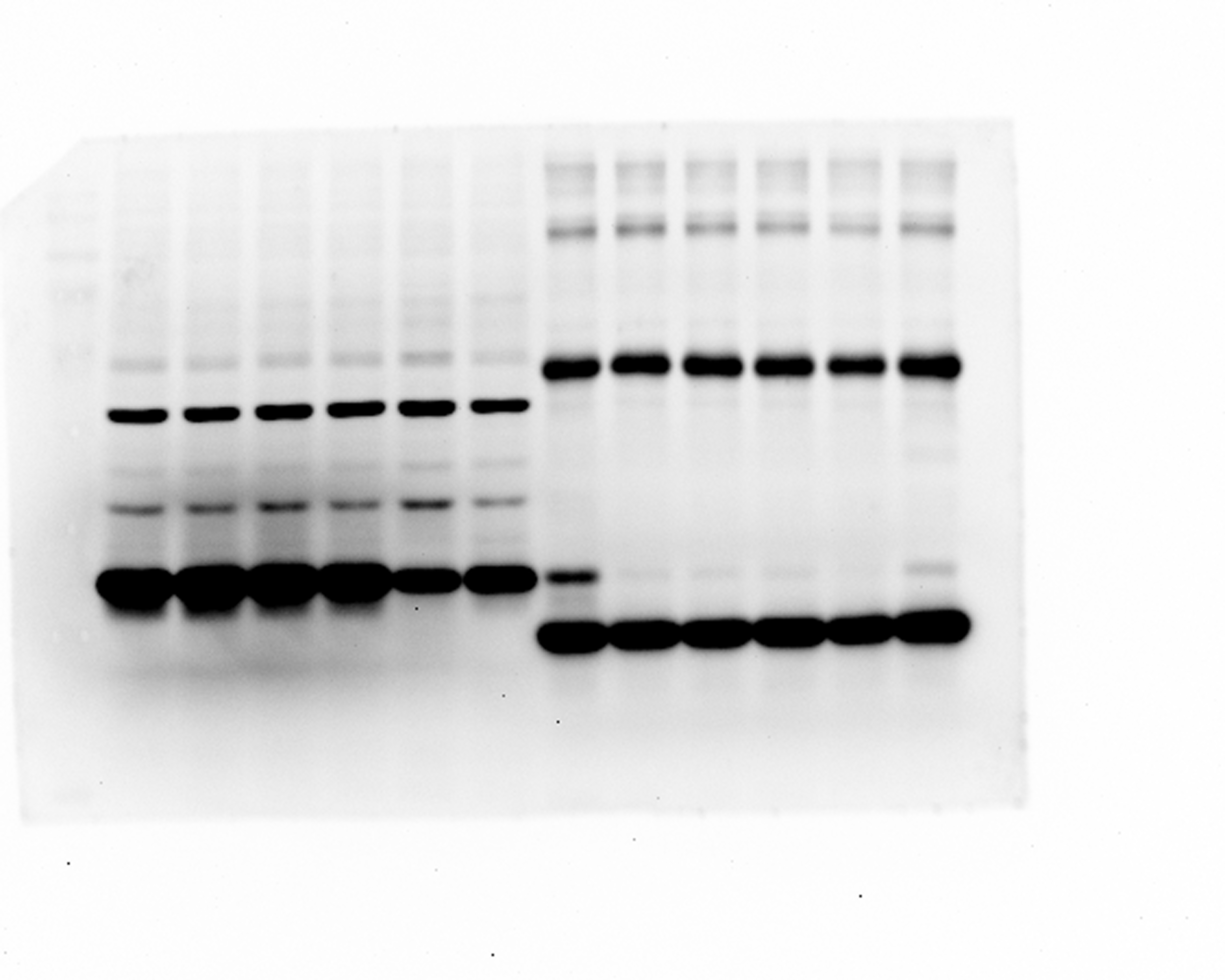

Supplement: Figure 8—source data 2. [file elife-81016-fig8-data2.zip › Rab11a-HA-Western blot/IP Raw file.png]

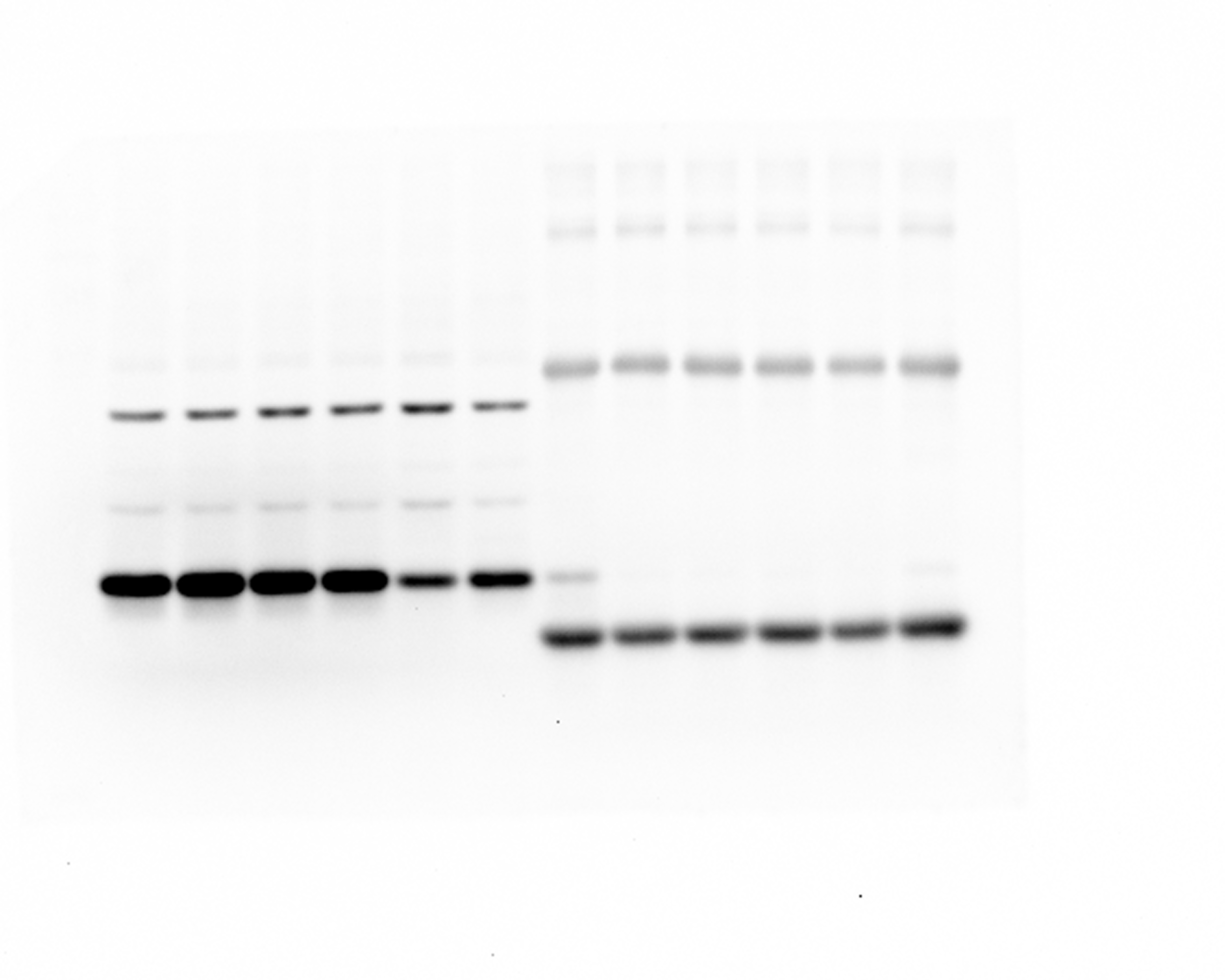

Supplement: Figure 8—source data 2. [file elife-81016-fig8-data2.zip › Rab11a-HA-Western blot/Lysate Raw file.png]
