# Supplementary material for: GJA1 depletion causes ciliary defects by affecting Rab11 trafficking to the ciliary base
Source: eLife. 2022 Aug 25;11:e81016. doi: 10.7554/eLife.81016 (PMC9448326; doi:10.7554/eLife.81016)
Supplement: Figure 7—figure supplement 3—source data 1. [file elife-81016-fig7-figsupp3-data1.zip › GJA1-Western blot/Figure.pptx]

## Slide 1
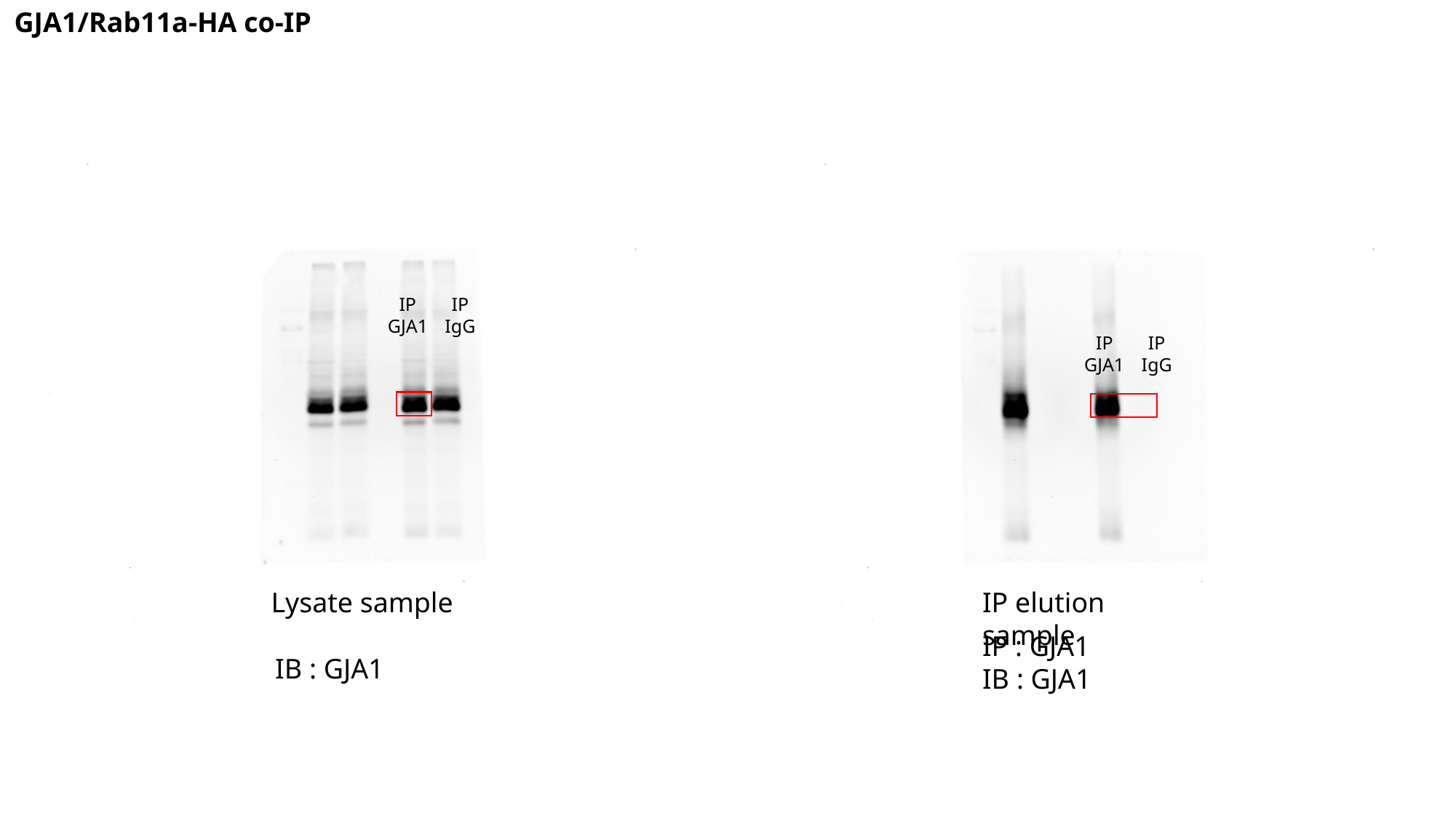

GJA1/Rab11a-HA co-IP
IP
GJA1
IP
IgG
IP
GJA1
IP
IgG
Lysate sample
IP elution sample
IP : GJA1
IB : GJA1
IB : GJA1
