# Supplementary material for: GJA1 depletion causes ciliary defects by affecting Rab11 trafficking to the ciliary base
Source: eLife. 2022 Aug 25;11:e81016. doi: 10.7554/eLife.81016 (PMC9448326; doi:10.7554/eLife.81016)
Supplement: Figure 8—source data 2. [file elife-81016-fig8-data2.zip › Rab11a-HA-Western blot/Figure.pptx]

## Slide 1
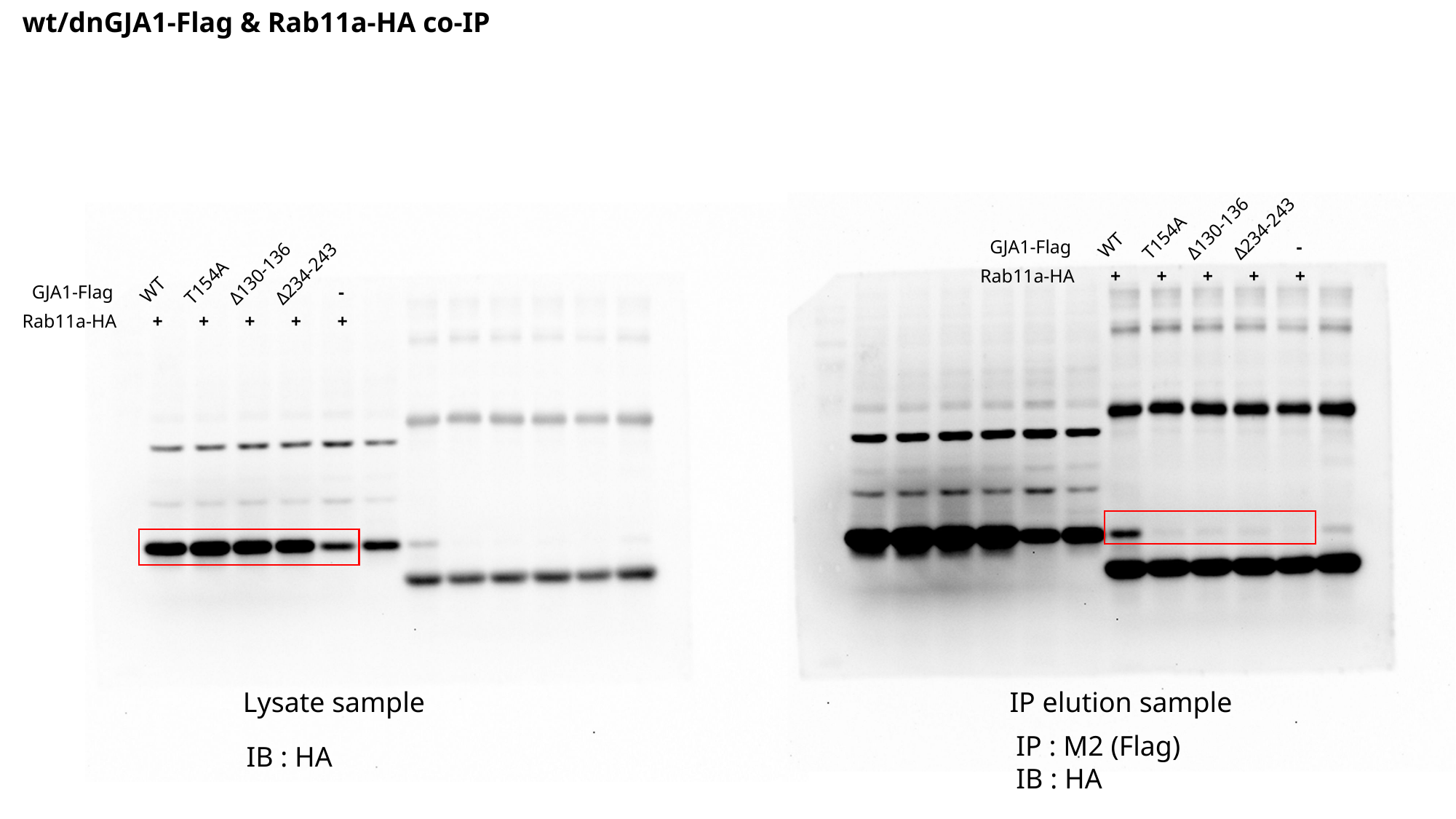

wt/dnGJA1-Flag & Rab11a-HA co-IP
Δ130-136
Δ234-243
T154A
WT
-
GJA1-Flag
Rab11a-HA
+
+
+
+
+
Δ130-136
Δ234-243
T154A
WT
-
GJA1-Flag
Rab11a-HA
+
+
+
+
+
Lysate sample
IP elution sample
IP : M2 (Flag)
IB : HA
IB : HA
